# Development of a Surgical Training Model for Pericardial Window in Cardiac Tamponade

**DOI:** 10.7759/cureus.90689

**Published:** 2025-08-21

**Authors:** Atsushi Tanikawa, Shoji Yokobori

**Affiliations:** 1 Department of Emergency and Critical Care Medicine, Tohoku University Hospital, Sendai, JPN; 2 Department of Emergency and Critical Care Medicine, Division of Neurosurgical Emergency, Nippon Medical School, Tokyo, JPN

**Keywords:** acute care surgery and trauma, emergency medicine physician, pericardial window, procedural skills training, skills and simulation training

## Abstract

Cardiac tamponade is a life-threatening condition that may require a surgical pericardial window. However, due to the rarity and urgency of such cases, opportunities for hands-on procedural training are limited.

We developed and evaluated a physical training model that simulates cardiac tamponade and allows for practice of the pericardial window procedure. The model was constructed using layered anatomical structures and simulated blood to replicate tamponade physiology. Emergency physicians and surgeons performed pericardiotomy using the model, after which a structured survey was conducted to assess its realism, educational value, and the participants’ confidence in performing the procedure.

Eight emergency or trauma physicians completed the procedure using the model and responded to the survey. The median participant age was 42 years, and three had no prior operative experience. The model received high ratings for both realism and educational value (median Likert score: 4).

This simulation model provides a realistic, reproducible, and portable platform for training in rare, high-acuity procedures without the need for live patients. It may help bridge the gap in emergency care and support curriculum development for surgical education.

## Introduction

Cardiac tamponade is an acute emergency that can rapidly lead to death without appropriate intervention [1,2]. Pericardiocentesis is commonly used, but in some cases, a surgical pericardial window is required. Due to the rarity of such cases and the technical difficulty, many emergency physicians have few chances to practice this life-saving procedure. To address this gap, we developed a simulation-based surgical training model to support pericardial window practice in a controlled environment.

## Technical report

**Figure 1:**
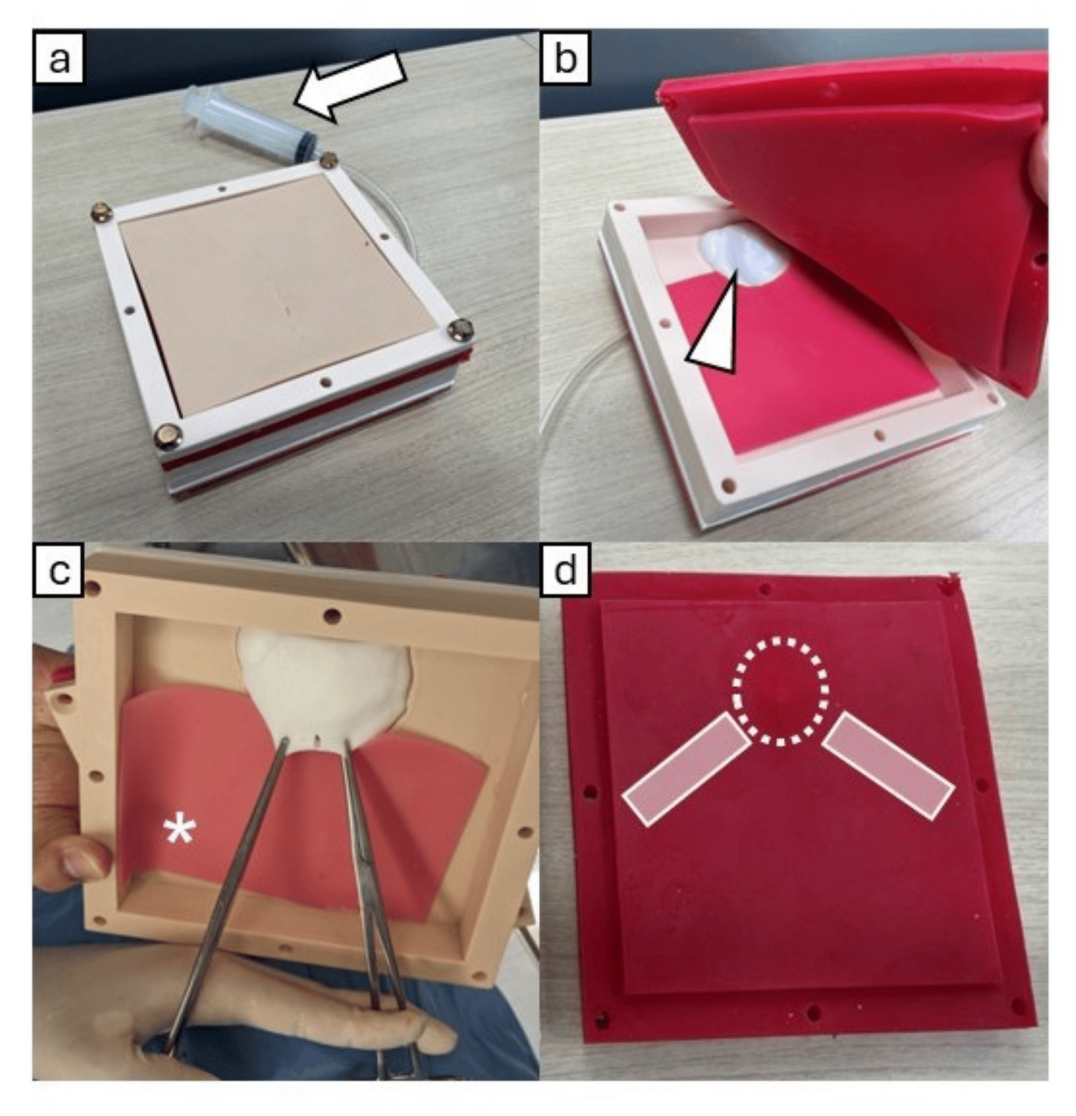
Construction of the Cardiac Tamponade Simulation Model a: The cardiac tamponade model. Simulated blood is injected into the pericardial sac using a syringe (white arrow). b: The pericardial sac (white arrowhead) is under the subcutaneous tissue, including the ribs and xiphoid. c: The pericardium is made of a material that does not tear when grasped by Kocher forceps, similar to the actual pericardium. The peritoneum in the model is composed of a thin membranous layer that lies in direct contact with the pericardium, replicating the anatomical relationship (asterisk). d. The xiphoid (dotted circle) and ribs (white rectangle) indicated within the subcutaneous tissue layer.

**Figure 2:**
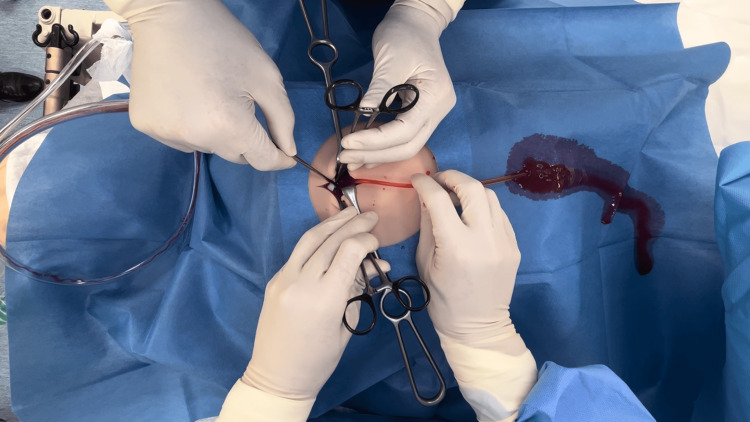
Simulation of Pericardial Window Procedure for Cardiac Tamponade Performing a pericardial window for cardiac tamponade model. After the pericardial window was created, a drain was placed, and the simulated blood was drained.

As part of a broader virtual reality-based educational project approved by the Institutional Review Board of Nippon Medical School Hospital (Approval No. B-2020-333), we developed a cardiac tamponade training model in collaboration with Kyoto Kagaku Co. (Kyoto, Japan) and Jolly Good Inc. (Tokyo, Japan). The model measures 175 mm (length) × 175 mm (width) × 55 mm (height) and includes hierarchical layers representing skin, subcutaneous tissue, ribs, xiphoid, pericardium, and heart (Figure 1). All components except for the frame are designed for single-use. Simulated blood is injected between the pericardium and heart to reproduce tamponade physiology (Figure 2). The material selection aimed to mimic the tactile feedback of real tissue.

Video 1 demonstrates the surgical performance of a pericardial window using the model of cardiac tamponade. 

**Video 1:** Surgical procedure of pericardial window using the cardiac tamponade training model. The left side of the screen corresponds to the patient’s head side. A subxiphoid skin incision was made to expose the xiphoid process, which was then removed. Initially, the peritoneum was mistakenly grasped with Kocher forceps instead of the pericardium. The pericardium was then identified and grasped with Kocher forceps. A pericardial window was surgically performed. Finally, a tube was inserted into the pericardial cavity.

Following model development, participants were recruited to evaluate the simulation. After completing the procedure in a controlled setting, they filled out a structured survey assessing the model’s realism, educational value, and their perceived confidence. Two structured questions were presented to participants. The first was, “Did the model help you practice the pericardial window?” The second was, “Do you feel capable of performing a pericardial window after practicing on this model?” Responses for both questions were rated on a 5-point Likert scale (1 = very poor; 5 = very good). Demographic data, prior experience, and subjective impressions were collected and analyzed descriptively.

Eight emergency or trauma physicians used the model to perform pericardial windows and subsequently completed a structured survey.

Participants' median age was 42 years (range: 39-53). On the question, "Did the model help you practice the pericardial window?" the median score was 4 (range: 4-5). For "Can you perform the procedure after practicing on this model?" the median was also 4. Notably, three participants had no prior operative experience with pericardial windows.

## Discussion

This physical model effectively simulates cardiac tamponade and the procedure for surgical drainage. Feedback from participants supports its realism and educational utility. Given the rarity of operative tamponade management in clinical settings, such a tool could bridge the training gap and improve procedural competence among emergency physicians [1,2].

Furthermore, the integration of virtual reality recordings alongside physical practice may enhance the learning experience by providing context, spatial orientation, and the opportunity for repeated observation. This dual-modality approach addresses both technical skill acquisition and cognitive preparedness. As the model does not require a live patient or high-fidelity simulation room, it is portable, reproducible, and scalable for widespread adoption in both resource-rich and limited settings [3,4].

Additionally, the structured feedback from participants - some of whom had no prior operative experience - indicates that this model can serve as a safe, confidence-building platform. Future directions could include multicenter studies, performance-based assessments, and incorporation into trauma or surgical simulation curricula. These steps may validate its effectiveness more broadly and support its role in credentialing or continuing medical education [5].

Simulation-based training becomes particularly valuable when targeting rare but life-threatening conditions, such as cardiac tamponade. Because clinicians may encounter such emergencies infrequently, yet require immediate and skilled responses, models like ours can serve as vital educational resources. Developing and disseminating realistic simulation tools for high-acuity, low-frequency scenarios should be a priority in advancing emergency care education [6,7].

## Conclusions

We developed a simulation-based training model for pericardial window procedures in cardiac tamponade, and preliminary evaluation suggests it may enhance procedural learning in emergency medicine. This aligns with efforts to advance simulation practice through high-fidelity, clinically relevant models.
